# Dopaminergic Neurones in the Main Olfactory Bulb: An Overview from an Electrophysiological Perspective

**DOI:** 10.3389/fnana.2017.00007

**Published:** 2017-02-14

**Authors:** Angela Pignatelli, Ottorino Belluzzi

**Affiliations:** Life Sciences and Biotechnology, University of FerraraFerrara, Italy

**Keywords:** dopaminergic neurones, olfactory bulb, electrophysiology, adult neurogenesis, experience-dependent plasticity

## Abstract

The olfactory bulb (OB), the first center processing olfactory information, is characterized by a vigorous life-long activity-dependent plasticity responsible for a variety of odor-evoked behavioral responses. It hosts the more numerous group of dopaminergic (DA) neurones in the central nervous system, cells strategically positioned at the entry of the bulbar circuitry, directly in contact with the olfactory nerve terminals, which play a key role in odor processing and in the adaptation of the bulbar network to external conditions. Here, we focus mainly on the electrophysiological properties of DA interneurones, reviewing findings concerning their excitability profiles in adulthood and in different phases of adult neurogenesis. We also discuss dynamic changes of the DA interneurones related to environmental stimuli and their possible functional implications.

## Introduction

Of the 11 distinct dopaminergic (DAergic) cell groups identified in the mammalian central nervous system (Dahlström and Fuxe, 1964; Felten and Sladek, 1983; Hökfelt et al., 1984; Dubach, 1994), the olfactory bulb (OB) hosts the most numerous (Guyenet and Crane, 1981; Cave and Baker, 2009) identified as A16 in the standard classification (Björklund and Dunnett, 2007).

Within the OB, DAergic neurones have been reported almost only in the most external (glomerular) layer (Halász et al., 1981), and it is generally accepted that they co-release dopamine (DA) and GABA from separate pools of vesicles (Maher and Westbrook, 2008; Borisovska et al., 2013). The glomerular layer is populated by a variety of cell types, essentially ascribable to three major classes of interneurones, i.e., periglomerular (PG), short-axon (SA) and external tufted (ET) cells. Since DA neurones are the only catecholaminergic neurones found in the OB (Kratskin and Belluzzi, 2003), they are usually recognized by the expression of tyrosine hydroxylase (TH), a rate-limiting enzyme of catecholaminergic pathway; it is estimated that 10%–16% of the neurones in the more external (glomerular) layer (GL) of adult animals are DAergic (McLean and Shipley, 1988; Panzanelli et al., 2007), and that they include two types of cells, PG (Kosaka et al., 1985; Gall et al., 1987) and a subpopulation of ET cells which, at difference from the tufted cells present in the external plexiform layer (EPL), are not projection neurones (Halász, 1990). Under the functional point of view, a first demonstration of the involvement of DA neurones in olfaction is provided by the impairment of olfactory discrimination in mice lacking functional dopamine receptors or transporters (Wilson and Sullivan, 1995; Tillerson et al., 2006; Taylor et al., 2009), and by the well-known observation that olfactory impairment is one of the earliest non-motor traits of Parkinson’s disease (Doty, 2012), preceding the onset of motor symptoms by years.

Bulbar DA cells are interesting under many aspects, but three characteristic in particular have attracted significant attention in the last years: first they are extremely plastic (Baker et al., 1983; Bastien-Dionne et al., 2010), second their position at the entry of the bulbar circuitry makes them obvious candidates for a significant role in the odor processing (Borisovska et al., 2013) and, third, DA cells are constantly generated throughout life (Altman, 1969; Betarbet et al., 1996; Baker et al., 2001; Winner et al., 2002; Mizrahi et al., 2006; Ventura and Goldman, 2007; Lazarini et al., 2014), an attribute that has raised further interest for the potentiality offered by neural stem cells in the germinal area (adult subventricular zone (SVZ)) as a source of autologous neuron for repopulation of the damaged areas in Parkinson’s disease (Cave et al., 2014).

Many excellent studies have covered the different aspects of DA neurobiology, especially for what concern the complex interplay of transcription factors, epigenetic control and role of non-neuronal cells in orchestrating birth, migration, differentiation and maintenance of these cells in adult neurogenesis (Saino-Saito et al., 2004; Hack et al., 2005; Kohwi et al., 2005; Brill et al., 2008; Havrda et al., 2008; Flames and Hobert, 2009; Cave et al., 2010; Caiazzo et al., 2011; Banerjee et al., 2013; Marei and Ahmed, 2013; Agoston et al., 2014; Vergaño-Vera et al., 2015; Bonzano et al., 2016; Rodríguez-Traver et al., 2016).

Here we rather focused on a somewhat lesser investigated aspect, the electrophysiological properties of adult born DA neurones in the different phases of their life, and the possible implication of their excitability profiles and adult neurogenesis in odor processing.

## Two Morphologically Distinct DA Neurones

The bulbar DA neurones have long been known to be different in size and morphology, presenting at least two main subtypes in almost all the species examined, including humans, (Halász et al., 1981; Davis and Macrides, 1983; Hoogland and Huisman, 1999; Pignatelli et al., 2005; Kosaka and Kosaka, 2007, 2008; Liberia et al., 2012). Several reviews address the analysis of the differences classifying the two DA interneurons subtypes, according to their morphological attributes and molecular signatures, in large vs. small (Kosaka and Kosaka, 2011; Imai, 2014; Nagayama et al., 2014). Kiyokage et al. (2010) propose an alternative classification in oliglomerular vs. poliglomerular DA neurones, suggesting that all DA interneurons are SA cells (Kiyokage et al., 2010)—we direct the reader wishing to learn more on this debate to the excellent recent review of Kosaka and Kosaka (2016). Given the demonstration that many DA PG cells exhibit the molecular markers of the presence of an axonal initial segment (IS), i.e., of an axon (Chand et al., 2015), we have adopted the first classification preferring, in this short review, to maintain the focus onto the less investigated problem of the functional properties of these cells.

In mice, the two main subtypes of OB DA neurones have average diameters of 8.76 ± 1.58 and 10.69 ± 2.70 μm (Kosaka and Kosaka, 2008) and membrane capacities of 5.41 ± 1.5 and 10.63 ± 3.45 pF (Pignatelli et al., 2005). Since the distributions of dimensions and membrane capacities of the two subtypes can be best fitted by two largely superposed Gaussian curves (see Figure 6 of Kosaka and Kosaka, 2007 and Figure 1C of Pignatelli et al., 2005), in most electrophysiological experiments it has not been possible to identify beyond any reasonable doubt the specific subtype of the cell recorded in OB slices. However, an interesting functional criterion for the discrimination of large vs. small DA neurones has been adopted by the group of M.S. Grubb, based on the observation that larger DA cells present an axon, whereas the smaller one are anaxonic (Chand et al., 2015; Kosaka and Kosaka, 2016). The action potentials (AP) originating in the soma and then propagating to the dendrites (soma-dendritic, SD AP) are slightly different from the AP originating in the IS and then back-propagating to the soma, where they are recorded (IS-SD AP); this barely noticeable difference in current-clamp recorded AP becomes more evident by representing the AP in a voltage vs. rise time plot (phase plane plot), where a noticeable bump in correspondence of the Hodgkin cycle initiation marks the IS-SD nature of the AP (Figure 2Dii of Chand et al., 2015). This criterion has been adopted to discriminate axonic-large vs. anaxonic-small DA cells in *in vitro* cultured dissociated cell (Chand et al., 2015; Galliano and Grubb, 2016) with remarkable results. Although the complement of voltage dependent channels in the two cell types does not appear to be significantly different (Pignatelli et al., 2005), the largest cells are more excitable than the smallest one, a disparity attributable to a series of differences in their excitability profile. In particular, the largest DA cells, with respect to the smallest one have lower threshold and rheobase current, faster rising phase of the AP, higher firing frequency, and other peculiarities discussed in detail in the recent article of Chand et al. (2015).

Larger TH+ neurones, initially regarded as ET cells (Halász et al., 1981; Davis and Macrides, 1983) and later recognized as GABAergic (Kosaka and Kosaka, 2007; Panzanelli et al., 2007; Parrish-Aungst et al., 2007), are glomerular interneurones giving a substantial contribution to interglomerular connections, establishing long-range intrabulbar coordination systems. A first intrabulbar association system has been described in which mirror-symmetric isofunctional odor columns (cross-laminar ensembles of neurones impinging onto a single glomerulus) are mutually connected through a reciprocal inhibitory circuit; the assembly includes distinct population of large TH+ ET cells making synapses on the granule cells on the opposite edge of the OB (Schoenfeld et al., 1985; Lodovichi et al., 2003; Kosaka and Kosaka, 2011). Another long-range intraglomerular association system sustained by large type DA glomerular neurones would connect glomeruli ipsilaterally (Kosaka and Kosaka, 2011). Accordingly, large DA neurones in the glomerular layer have been shown to express axon initial segment (AIS) markers, absent in the small DA neurones, both *in vivo* (Kosaka and Kosaka, 2011) and *in vitro* (Chand et al., 2015; Galliano and Grubb, 2016).

Small TH+ cells, accounting for about 85% of the bulbar DA neurones (Pignatelli et al., 2005), appear to extend their connection to a single glomerulus, or to few close glomeruli (Kosaka and Kosaka, 2008, 2011, 2016).

Differences among large- and small-sized TH+ glomerular cells are also observed in the adult neurogenesis: the investigation of their birth dates has shown that large DA neurones are born only pre- and perinatally, never in adulthood, whereas the small type of DA neurones are generated also in adult periods (Kosaka et al., 1987; Vergaño-Vera et al., 2006; Bovetti et al., 2009; Kosaka and Kosaka, 2009; Galliano and Grubb, 2016).

## Electrophysiology of Mature Bulbar DA Neurones

DA-PG cells in the OB have been the object of electro-physiological studies (Pignatelli et al., 2005; Puopolo et al., 2005), which have provided a comprehensive description of the complex system of the voltage-dependent conductances determining their excitability profile, including the pacemaking machinery.

DA bulbar neurones present two large and five small voltage-dependent conductances; all of them have been kinetically characterized, and an Hodgkin-Huxley computational model of DA PG cells has been elaborated (Pignatelli et al., 2005). The two conductances having the largest amplitude, responsible for the generation of the action potential, are a fast transient sodium current (101 nS) and a delayed rectifier potassium current (50.1 nS; Pignatelli et al., 2005)—no other K^+^ currents, as I_A_, present in immature DA PG cells (see below) and in other mature PG cells subtypes (Fogli Iseppe et al., 2016) have been reported.

As most DA neurones, bulbar DA cells have a spontaneous activity, primarily within the theta frequency range (4–12 Hz); the pacemaking machinery is composed of a system of two small inward currents, a persistent sodium current (I_Na(P)_; 0.41 nS) and a T-type Ca^2+^ current (I_Ca(T)_; 0.35 nS; Pignatelli et al., 2012).

In addition to TTX, I_Na(P)_ can be selectively blocked by riluzole (Urbani and Belluzzi, 2000), a drug used in the therapy of amyotrophic lateral sclerosis, and indeed riluzole (5 μM) completely suppresses spontaneous firing.

The T-type calcium current is blocked by low concentrations of nickel (50–100 μM; Lee et al., 1999), and by mibefradil (Mishra and Hermsmeyer, 1994), and both reversibly block spontaneous activity (Pignatelli et al., 2005).

Based on the kinetic data, a numerical reconstruction of the bulbar DA neurones according to the Hodgkin-Huxley model (Hodgkin and Huxley, 1952) has been developed (Pignatelli et al., 2005), which incorporates all the conductances detected and assuming the cell as a single electrotonically compact compartment. The *in silico* tests made possible by the model can be very helpful to uncover the essentials of the relative contribution of the currents sustaining the pacemaking process and their reciprocal interactions. The main outcomes obtained from the *in silico* cell model can be recapitulated as follows:

- the numerical cell model is capable to fire spontaneously at the same frequency of real neurones.
- the *primum movens*, the current which first sets in motion the process causing the progressive depolarization of the cell during the interspike interval, is the I_Ca(T)_, replaced by I_Na(P)_ in the second half of the slow depolarizing phase, until the threshold for the fast Na-current is reached and the action potential develops. Both currents are amazingly small in amplitude (max 4 pA) compared with sodium and delayed rectifier potassium currents underlying the action potential (about 1 nA) but, nevertheless, they are sufficient to depolarize DA PG cells, due to the high input resistance of these cells (about 700 MΩ).
- in the numerical model—as in real preparations—both I_Na(P)_ and I_Ca(T)_ are required to sustain spontaneous activity, as the selective block of one or both of them abolishes the spontaneous firing: the *in silico* cell, as DA neurones, responds with a single spike to a depolarizing current pulse, but is unable to fire spontaneously when I_Na(P)_ and I_Ca(T)_ are zeroed.
- the model indicates that the T-type calcium channels are decisive in determining the firing frequency as small changes in I_Ca(T)_ conductance (from 0.35 nS to 0.4 nS) suffice to change the spontaneous firing frequency from 8 Hz to 16 Hz.
- the high voltage-activated calcium channels are not required for the pacemaking mechanism, and their blockage, both in living DA neurones and in the model, has no effect on the spontaneous firing frequency.

Two other small conductances, activated by hyper-polarization, are present in bulbar DA cells, not directly involved in the pacemaking machinery but playing an important role in its modulation: an h-current (Fried et al., 2010; Pignatelli et al., 2013) and a potassium inward rectifier (KIR) current (Borin et al., 2014). Both currents are active at rest, and exert opposite effects on the resting membrane potential, depolarizing the h-current and hyperpolarizing the KIR, governing the resting membrane potential and consequently exerting an important role in controlling the excitability of these cells.

Both hyperpolarization-activated currents are effectively modulated by second messenger mechanisms, and DA neurones in the OB receive numerous afferents releasing a multiplicity of neurotransmitters, in many cases capable of affecting the cAMP pathway, and therefore potentially capable of modulating both h- and KIR currents. To name only a few, the OB receives serotoninergic afferents from the ventral and dorsal raphe nuclei (Araneda et al., 1980), noradrenergic input from the *locus coeruleus* (McLean et al., 1989), cholinergic inputs from the nucleus of the horizontal limb of the diagonal band (Zaborszky et al., 1986), and histaminergic inputs from hypothalamus (Panula et al., 1989). Accordingly, the KIR current is under the influence of a multiplicity of molecular pathways, which can either enhance the current, as it happens with D2, muscarinic, and GABA_A_ receptor agonists, or have the contrary effect, as it is observed with α1, 5-HT and histamine receptor agonists (Borin et al., 2014). Contrary to the KIR, the h-current seems to be modulated only by a single neurotransmitter, noradrenaline, which has a profound inhibitory influence on the current (Pignatelli et al., 2013). Taken together, these characteristics of the two currents activated by hyperpolarization provide the basis for a multiplicity of modulatory mechanisms converging onto DA-PG cells, making them fully qualified to reconfigure the bulbar network for better flexibility.

## Adult Neurogenesis

OB interneuron progenitors in mice generate from neural stem cells in the SVZ of the lateral ventricle (for recent reviews see Cave and Baker, 2015; Lledo and Valley, 2016; Malvaut and Saghatelyan, 2016). The adult SVZ can be subdivided into several domains identified by the expression of diverse transcription factors, highly conserved (Fujiwara and Cave, 2016). These domains produce neuroblasts committed to differentiate into distinct subsets of OB interneurones—DA progenitors are generated from the dorsolateral region (for a review see Fiorelli et al., 2015), characterized by the expression of the transcription factor Pax6 (Merkle et al., 2007; Young et al., 2007; Brill et al., 2008; Fernández et al., 2011). Pax6 is required for the development of the DAergic phenotype (Dellovade et al., 1998; Kohwi et al., 2005; Brill et al., 2008; Haba et al., 2009), and operates in association with the Dlx2 and Meis2 transcription factors (Brill et al., 2008; de Chevigny et al., 2012; Agoston et al., 2014). For its distinctive expression in terminally differentiated DAergic neurones and its requirement for their survival (Ninkovic et al., 2010), Pax6 can be considered almost a hallmark of bulbar DA neurones.

The rate of production of bulbar DA neurones is generally reported to increase in the postnatal/adult OB (Kosaka et al., 1987; McLean and Shipley, 1988; Winner et al., 2002; De Marchis et al., 2007, but see also Batista-Brito et al., 2008). Using *in vivo* imaging and genetic fate-mapping techniques, the fate of adult-born neurones has been traced over up to 9 months (Ninkovic et al., 2007; Adam and Mizrahi, 2011). The situation appear to be different in granule and glomerular layer: whereas in the granule cell layer after the second month the whole population of granules remain numerically stable, indicating that substantially there is only a turnover of this type of cells, in the glomerular layer the addition of new neurons to the adult bulbar network outnumbers by about 30% the cell loss in the glomerular layer (Ninkovic et al., 2007). Interestingly, this net addition does not concern all the adult-born cell of the GL, but only two subtypes, calretinin and DA interneurones (Ninkovic et al., 2007; Adam and Mizrahi, 2011), suggesting that the adult neurogenesis in the OB is subtype-specific, and regulated differently in granule and glomerular layer.

Several studies have shown that survival and integration of adult-born neurones in the OB critically depend on the fullness of the olfactory input, both processes being strongly enhanced by odor enrichment (Rochefort et al., 2002; Yamaguchi and Mori, 2005; Bonzano et al., 2014)—see also below. DA PG cells are the only bulbar interneurones receiving direct input from the olfactory nerve (Kosaka and Kosaka, 2007), and therefore it is not surprising that they are particularly sensitive to the level of olfactory input, which controls dynamically the turnover in a spatial and neuronal subtype-specific manner (Sawada et al., 2011).

As indicated above, in this context we will limit the discussion on the electrophysiological aspects of this process.

## Electrophysiology of DA Neurones during Adult Neurogenesis

Although mature DA neurones within the OB are expressed almost exclusively in the more external (glomerular) layer (Halász et al., 1981), spare neurones expressing the eGFP under the TH promoter have been observed also in the EPL, a neuropil-rich area positioned between the mitral and glomerular layers, and in a narrow region encompassing mitral and internal plexiform (M/IP) layers; these are cells in which the transcription of the TH gene occurs in the absence of significant translational activity (Baker et al., 2001; Jeong et al., 2003).

The presence of TH transcription in cells lying in a region devoid of DA neurones has been proposed to be ascribable to adult neurogenesis: the cells expressing TH but not DA could be newly generated neurones committed to a DA phenotype, just arrived in the M/IP following the rostral migratory stream (Saino-Saito et al., 2004), which could represent different stages of maturation of DA neurones. Since immature DA neurones have distinct physiological signatures, this hypothesis has been tested with targeted electrophysiological recordings at different levels within the OB (Pignatelli et al., 2009) using TH-GFP transgenic mice (Sawamoto et al., 2001); in immature DA neurones, the transcription of the TH gene is not immediately followed by translation (Baker et al., 2001), but in these neurones the eGFP gene, located under the same promoter, undergoes a certain degree of translation, small, but sufficient to allow these cells to become noticeably—although dimly—fluorescent.

TH-GFP neurones in the external plexiform and mitral/internal plexiform layers show very different excitability profiles. The TH-GFP cells of the EPL present the same set of voltage-dependent currents as for mature DA neurones of the glomerular layer, including fast transient and persistent Na-currents, T-type and L-type Ca-currents, and delayed rectifier potassium; accordingly, their behavior, irrespective of their position in the layer, is virtually identical to that of mature DA neurones in the glomerular layer, showing a spontaneous firing almost indistinguishable from that of the mature DA neurones of the GL (Pignatelli et al., 2009).

The mechanisms governing autorhythmicity of TH-GFP cells in external plexiform and glomerular layers are essentially the same: as for mature DA neurones, TH-GFP neurones in the EPL display a persistent Na-current (I_NaP_) and a T-type calcium current, and the pharmacological block of any of them also reversibly blocks the spontaneous activity (Pignatelli et al., 2012).

The appearance of the T-type calcium channel during maturation of TH-GFP cells has been studied by analyzing the expression of the calcium channel gene CAV3.2 in different groups of cells separated with fluorescent activated cell sorting (FACS) according to the intensity of their fluorescence (a value indicative of the maturation of the cell); the gene CAV3.2 has been selected for the sensitivity of the calcium channel encoded by this gene to low nickel (Lee et al., 1999). The results indicate a strong correlation (fivefold increase) between cell maturation and CAV3.2 mRNA levels, suggesting that the transcription of the gene coding for this channels is strongly associated with the process of maturation of DA neurones (Pignatelli et al., 2009).

Further information has been obtained from the analysis of [Cl^−^]_i_ in developing DA cells. Although it is the main inhibitory neurotransmitter in the mature brain, in the earlier phases of development GABA is excitatory, depolarizing the neurones by promoting an outflow of Cl^−^ ions, as result of the unusual balance between the cation-chloride importer NKCC1 and the extruder KCC2; in newly generated cells this is dominated by the importer, leading to accumulation of Cl^−^ inside the cell and to Nernstian equilibrium potential for Cl^−^ ions positive with respect to the resting membrane potential (Ben-Ari et al., 2007). The analysis of [Cl^−^]_i_ in TH-GFP cells shows that this progressively decreases moving from M/IP to GL layers, and this observation is paralleled by an increase of the ratio extruder vs. accumulator in the same axis (Pignatelli et al., 2009).

The last observation concerning the maturation of DA neurones resulting from adult neurogenesis is about the establishment of synaptic connections with the existing network. Two well-established properties of mature DA neurones in the GL is that the large majority (about 80%) of the afferent synapses are asymmetrical from olfactory nerve fibers (Toida et al., 2000) and that the full expression of the DAergic phenotype necessitates a well-structured input from olfactory receptor cells (Brunjes et al., 1985; Stone et al., 1991; Wilson and Wood, 1992; Baker et al., 1993; Cho et al., 1996).

The majority (75%) of the TH-GFP cells in the EPL respond to ON stimulation with monosynaptic EPSP which can be reversibly suppressed by kynurenate (Pignatelli et al., 2009). On the contrary, the TH-GFP cells in the M/IP layer do not respond synaptically to the ON stimulation but are depolarized in response to focal application of glutamate, showing that they already have functional glutamate receptors (Pignatelli et al., 2009).

In this hypothesis, the faintly fluorescent cells observed in the mitral and internal plexiform layers could represent elements having arrested their migration process at this level, conceivably waiting for some consensus clue to come from the glomerular region allowing them to attain their final destination moving across the EPL while finalizing their differentiation towards the DAergic phenotype.

In a recent article, using a long-term *in vivo* single-cell tracking based on a newly developed optical cell positioning system, a series of remarkable new observations have been reported concerning the movements of neuroblasts once arrived in the OB (Liang et al., 2016). Of particular interest in this context, is the observation that some of the neuroblasts bound to the GL, “stopped for very long periods (from 12 h to a few days) before resuming the movement” (Liang et al., 2016), then crossed rapidly the EPL and once reached the GL switched to lateral movement, eventually integrating few glomeruli away from their entry point in the GL, and it is tempting to envisage that the cells showing this behavior could be DA neurones.

## Experience-Dependent Plasticity in Adult Neurogenesis

A hallmark of bulbar DA neurones is their strong plasticity in response to sensory stimuli at multiple levels.

A first level of odor-driven plasticity observed in DA cells consists in their modulation by the olfactory input: sensory activity is fundamental for the development and maintenance of DA, but not of GABAergic, calretinin, or calbindin phenotypes, suggesting that DA neurones have a distinct reliance on odor-induced activity, marking a significant difference with respect to the other PG cells. DA neurone density is strongly and reversibly down-regulated in animals odor deprived following either chemical or surgical deafferentation of the OB (Nadi et al., 1981; Kawano and Margolis, 1982; Baker et al., 1983), or naris occlusion (Baker et al., 1983; Brunjes et al., 1985), an effect which applies to both pre-existing and adult-generated neurones (Bovetti et al., 2009; Bastien-Dionne et al., 2010). A drastic reduction of DA neurones ensues rapidly (4 days) the loss of sensory input (Baker et al., 1983), then proceeds more gradually, reaching a maximal 40% loss after 4 weeks (Sawada et al., 2011); although this phenomenon has long been known, the underlying molecular mechanisms are only beginning to emerge, and include transcriptional and epigenetic regulators (Banerjee et al., 2013; Bovetti et al., 2013) and microglia (Grier et al., 2016).

A second level of sensory-dependent plasticity of bulbar DA neurones involves environmental modulation of adult neurogenesis. In mice, an odor-enriched milieu has been shown to affect both adult neurogenesis and learning, an effect which is specific because it does not influence hippocampal neurogenesis (Rochefort et al., 2002), and it has been shown that this effect selectively affects the DA neurones, due to increased neurogenesis, whereas similar changes in calretinin or calbindin neurones were not observed (Bonzano et al., 2014), but see also (Kato et al., 2012). Odour enrichment or deprivation also increases or decreases, respectively, the survival of adult-born glomerular neurones, including DA cells (Bovetti et al., 2009), and odor deprivation upregulates the critical transcription factor Pax6 is in mice bulbar TH-positive cells (Bastien-Dionne et al., 2010).

A further level of sensory-dependent plasticity involves an activity-dependent upregulation of synaptogenesis of adult-born PG cells, also at the level of dendritic spines, and particularly prominent during their initial phases of development (Kelsch et al., 2009; Livneh et al., 2009); interestingly, plastic patterns of synaptic connectivity associated with learning has also been observed in adult-born granule cells (Breton-Provencher et al., 2016; Huang et al., 2016).

In this context, an interesting problem concerns the timing of DA neurogenesis and the functional inclusion of newborn neurones in the bulbar network. There is a general consensus on the principle that adult neurogenesis adds an essential degree of freedom to odor processing by adapting the OB circuitry to new olfactory signals or situations, but how could this be possible if it takes not less than 2 weeks to produce new neurones which are completely functionally integrated (Ortega-Perez et al., 2007)? In other words, how can the addition of new elements to the neuronal network be the response to an unexperienced functional context if the rewiring will only be realized well after the triggering stimulus is over? A similar objection was originally raised for neurogenesis in hippocampus (Kempermann, 2002).

The seminal observations on the phenotypic differentiation of DA progenitor cells in the OB put forward by the Harriett Baker group (Baker et al., 2001), suggest a different way to look at the problem: new cells are incessantly produced, complete their tangential migration to the OB, and begin their differentiation process toward their final phenotype. They halt their radial migration halfway in the mitral cell layer, where they suspend their maturation process in a pre-terminal state, waiting for a consensus signal, which will allow them to perfect their differentiation towards the final phenotype and to take their place within the bulbar circuitry. What this consensus signal might be is not known, but the data from our group outlined above suggest that it follows the formation of a synaptic contact with the olfactory nerve. What we propose is that at any given moment there are new cells in the mitral cell layer committed to a DA phenotype but not fully mature sending their projection into the glomerular layer and trying to establish synaptic contacts. Failing to do so, the newly generated cell undergo apoptosis and die, what is actually known to be the fate of the majority of newborn cells reaching the OB (Biebl et al., 2000; Winner et al., 2002). If, on the contrary, an operative synaptic contact can be set up, possibly with a spine relocation process of the kind described in adult-born granule cells (Breton-Provencher et al., 2016), then the progenitor neurone completes its differentiation process migrating to its ultimate destination. Such a process might explain how the OB circuitry could rapidly adjust to better tackle new stimuli from the outside world, optimizing its wiring for a more effective signal processing. With a mechanism of this kind, the delay between a new sensory experience and the circuit modifications for optimal signal processing would be extremely small, as rewiring would only require the refinement and a short-range repositioning of plastic elements already present *in situ*, not a *de novo* production in a remote site.

## Role of DA Neurones in the Olfactory Bulb

At cellular level, the data of the literature about the role of DA neurones in the bulbar circuits are of uncertain interpretation. To limit the discussion to what has received ample experimental support, a role of DA in the OB is the inhibition of glutamate release in olfactory sensory fibers, via activation of D_2_ presynaptic receptors (Wachowiak and Cohen, 1999; Berkowicz and Trombley, 2000), activating an intracellular pathway suppressing of calcium influx through N-type calcium channels (Wachowiak et al., 2005). The autorhythmicity of PG DA neurones results in a tonic release of neurotransmitter in the synaptic cleft between DAergic cells from ON terminals, as shown by a ~30% increase in levels of intracellular Ca^2+^ following the blockage of D_2_ receptors (Wachowiak and Cohen, 1999). DA can be eliminated from the synaptic cleft following a reuptake by the DA transporter (DAT) or by the breakdown enzyme catechol-O-methyl-transferase (COMT). A recent study of Cockerham et al. (2016) has examined the mechanisms of DA clearance in the synaptic space, showing that, contrary to what happens in the striatum, where the reuptake is mainly driven by the first, the predominant mechanism for DA clearance in the OB is the COMT breakdown. The authors suggest that the combined provisions of activity-dependent DA levels and activity independent COMT breakdown can extend the dynamic range of olfactory afferents in specific glomerular circuits (Cockerham et al., 2016) and/or could create adaptive odorant-specific filters for sensory inputs (McGann, 2013).

Larger DA neurones (a.k.a. SA cells), making interglomerular connections, establish synaptic contacts with ET cells, which in turn make direct excitatory synapses onto projection neurones (M/T cells). Using an optogenetic approach, the synapse between SA and ET cells has been characterized, showing that it elicits a biphasic response in ET cells with an initial GABA_A_ receptor-mediated monosynaptic inhibition, followed by D_1_ receptor-mediated excitation (Liu et al., 2013). The integration of the two responses is made by the h-current: an initial GABA_A_ receptor-mediated a hyperpolarization, activating an h-current, is immediately followed by the action of the co-released DA, which potentiates the I_h_, with a consequent depolarizing rebound of the membrane potential (Liu et al., 2013). The net result of the biphasic response observed in ET cells is that it could act as a gate in the transmission of sensory signals to projecting neurones (Banerjee et al., 2015), influencing the processing of sensory input with a mechanism that could be further tuned by the regulation of GABA vs. DA action by COMT (Cockerham et al., 2016).

At a functional level, the contribution of mature and/or newborn DA neurones in odor processing remains substantially elusive, although a certain number of considerations can be made, derived from direct experimental investigation or by analogy with other DA systems.

To begin with, it is not clear the contribution of DA neurones in odor detection, as mice knockout for D2 receptors have an almost normal capability to detect or discriminate odors; however, their ability to elaborate a correct response to unpredicted odor-driven contingencies or situations is significantly impaired (Kruzich and Grandy, 2004). In behavioral terms, this could be described a severe impairment in reversal learning, a measure of cognitive flexibility (Izquierdo et al., 2016), and therefore a kind of processing which is likely to take place in circuits higher than the OB.

More focused on the role of DA cells in bulbar circuitry is a recent study, confirming an earlier observation (Davila et al., 2003), which demonstrates that DA cells, via ET cells—to which they are coupled with chemical and electrical synapses—inhibit mitral and tufted cells, thereby controlling the gain and decreasing the correlation of odor images in projection neurones (Banerjee et al., 2015).

In accordance with the key function attributable to DA cells for their strategic position at the entry of the bulbar circuitry, several studies have suggested a major role of the DA system in specific aspects of odor processing and of odor-driven behaviors, to cite only a couple odor discrimination and reproduction.

As already outlined above, the DAergic system is involved in odor discrimination rather than in odor detection (Kruzich and Grandy, 2004). Increasing of DA levels by injecting the dopamine precursor L-DOPA in rats improves olfactory discrimination capabilities in forced choice odor discrimination task (Pavlis et al., 2006), and mice lacking the DAT show discrimination deficits (Tillerson et al., 2006). The mechanism is mediated by modulation of D2 receptors and affects discrimination capabilities by altering the perceived odor intensity (Doty and Risser, 1989; Wei et al., 2006).

Reproduction sees an important participation of the DAergic system in different moments, from mating to offspring recognition. Aftermating odor perception is associated with a surge of TH expression in PGCs and DA transmission in the OB (Serguera et al., 2008). This rise in DA release translates into inhibition of ON terminals and downturn of sensory input and excitation of the OB projection neurones, eventually blocking social olfactory cues detrimental to pregnancy (Serguera et al., 2008). DA levels increase significantly in the OB during parturition and suckling (Kendrick et al., 1988), deeply influencing the maternal behavior (Keverne et al., 1993).

## Concluding Remarks

Although bulbar DA cells have been the object of many studies covering their histological and electrophysiological profiles, it is amazing that the functional role of what is the largest population of DAergic neurones in the brain remains in the shadow under many aspects, and this is particularly true for adult-born DA cells. Understanding how DA neurones contribute to signal processing in the bulbar network requires a finer knowledge of their connections, not as much under the anatomical aspect as under the dynamic aspect, and of their “molecular” (Bhalla, 2014) computational capabilities. New technical approaches are progressively revealing new levels of complexity in the computational capabilities of these cells, and in the variety of the roles they can play, and we can expect interesting developments in the incoming years.

Adult neurogenesis of DA neurones also in humans (Inta et al., 2015), demonstrating the capacity of the mature CNS to regenerate cells whose loss is responsible for devastating neurodegenerative diseases, has turned on hopes that understanding the mechanisms governing adult neurogenesis could promote new strategies for cell replacement therapies, either by implementing the endogenous neurogenic potential or by transplants of highly neurogenic stem cell supplies. To date, despite the considerable amount of information accumulated on adult neurogenesis in the course of the last 20 years, no significant translational improvements have been achieved for most neurological diseases, including those more directly linked to a damage of the DA system, and the translational gap remains wide open (Cattaneo and Bonfanti, 2014), but a reasonable possibility that filling this gap is not just a hope do exist; the years to come might substantiate this expectation, and the DAergic neurones of the OB might be a main character in the play.

## Author Contributions

OB: MS drafting; AP: bibliographic research.

## Funding

This work was funded by the University of Ferrara, FAR 2015 program to OB.

## Conflict of Interest Statement

The authors declare that the research was conducted in the absence of any commercial or financial relationships that could be construed as a potential conflict of interest.
